# Structural and Molecular Characterization of Squalene Synthase Belonging to the Marine Thraustochytrid Species *Aurantiochytrium limacinum* Using Bioinformatics Approach

**DOI:** 10.3390/md20030180

**Published:** 2022-02-28

**Authors:** Sachin Vyas, Maurizio Bettiga, Ulrika Rova, Paul Christakopoulos, Leonidas Matsakas, Alok Patel

**Affiliations:** 1 Biochemical Process Engineering, Division of Chemical Engineering, Department of Civil, Environmental, and Natural Resource Engineering, Luleå University of Technology, 97187 Luleå, Sweden; sachinkumar.rajendrakumar.vyas@ltu.se (S.V.); ulrika.rova@ltu.se (U.R.); paul.christakopoulos@ltu.se (P.C.); leonidas.matsakas@ltu.se (L.M.); 2 Department of Biological Engineering, Chalmers University of Technology, 41296 Gothenberg, Sweden; maurizio.bettiga@chalmers.se; 3 Bioeconomy Division, EviKrets Biobased Processes Consultants, Lunnavågen 87, 42834 Landvetter, Sweden

**Keywords:** marine thraustochytrids, *Aurantiochytrium*, squalene, squalene synthase, bioinformatic analysis

## Abstract

The marine microorganisms thraustochytrids have been explored for their potential in the production of various bioactive compounds, such as DHA, carotenoids, and squalene. Squalene is a secondary metabolite of the triterpenoid class and is known for its importance in various industrial applications. The bioinformatic analysis for squalene synthase (SQS) gene (the first key enzyme in the tri-terpenoid synthesis pathway), that is prevailing among thraustochytrids, is poorly investigated. In-silico studies combining sequence alignments and bioinformatic tools helped in the preliminary characterization of squalene synthases found in *Aurantiochytrium limacinum.* The sequence contained highly conserved regions for SQS found among different species indicated the enzyme had all the regions for its functionality. The signal peptide sequence and transmembrane regions were absent, indicating an important aspect of the subcellular localization. Secondary and 3-D models generated using appropriate templates demonstrated the similarities with SQS of the other species. The 3-D model also provided important insights into possible active, binding, phosphorylation, and glycosylation sites.

## 1. Introduction

Squalene (a triterpene), a potential antioxidant, is a bio-product in high demand due to its application in various industries, such as cosmetics, pharmaceuticals, and food industries [1,2,3]. Many deep-sea sharks have been hunted for a long period for the extraction of squalene [4,5]. Apart from other sources, such as plants and chemical synthesis, researchers are focusing on the development of microbial factories for the commercial production of squalene [6]. Various microorganisms, such as yeast and microalgae, have been explored [7,8]. Among all, marine heterotrophic microorganisms, such as thraustochytrids, are promising sources of squalene [2,3,9]. *Aurantiochytrium* species among many thraustochytrids has been the center of interest and promising for the production of squalene at commercial level (up to 30% of cell dry weight) [3,10,11].

There are two major pathways for terpenoid synthesis: (i) The MVA (mevalonate) pathway and (ii) the MEP (2-C-methyl-d-erythritol-4-phosphate) pathway [12,13]. Squalene synthesis in thraustochytrids is believed to follow the MVA pathway [14]. Squalene synthase (EC 2.5.1.21; SQS) is the first rate-limiting enzyme of the reaction of the squalene synthesis in thraustochytrids. Two molecules of farnesyl pyrophosphate (FPP) resulting from the acetyl-CoA via subsequent multi-step reaction are utilized to produce squalene via the catalytic action of squalene synthase [14]. The mechanism of SQS has been well characterized as in two steps (1) condensation: Pre-squalene diphosphate (PSPP) generation from two molecules of farnesyl diphosphate (FPP) and (2) squalene production from PSPP in the presence of NADPH and Mg^2+^ [15]. The effect on squalene and other secondary metabolite production by overexpression and disruption of squalene synthase in various organisms has been extensively studied [2,14]. Thus, the bioinformatic analysis for the sequences available from genomes of thraustochytrids will aid in designing future enhanced squalene or carotenoids synthesis.

The physicochemical prediction, detailed bioinformatics analysis, and 3-D structure of the SQS enzyme in any thraustochytrid species have not been fully reported yet [2]. The genome sequences obtained for *Aurantiochytrium limacinum* reveal that SQS is very important for overall terpenoid synthesis. The key features and information of the SQS like potential active sites, phosphorylation sites, etc., will be helpful in rational designing of the genetic engineering experiment in the future to improve squalene production [2]. In the present studies, for the first time, in-silico analysis for the SQS gene found in thraustochytrid *Aurantiochytrium limacinum* species was performed. A detailed analysis of the conserved regions and important domains responsible for binding substrate and ligands was studied. The secondary and 3D structure for the SQS in the selected species was designed using appropriate known structures of the enzyme (Human SQS) as a template.

## 2. Results and Discussions

### 2.1. Conserved Domain Search and Multiple Sequence Analysis

**Figure 1:**
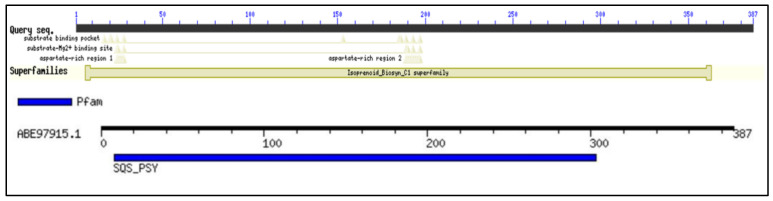
Conserved domains and motifs for the SQS genes of *Aurantiochytrium limacinum*: Showing the conserved motifs, superfamily, substrate binding, and aspartate rich regions for the selected sequence.

**Figure 2:**
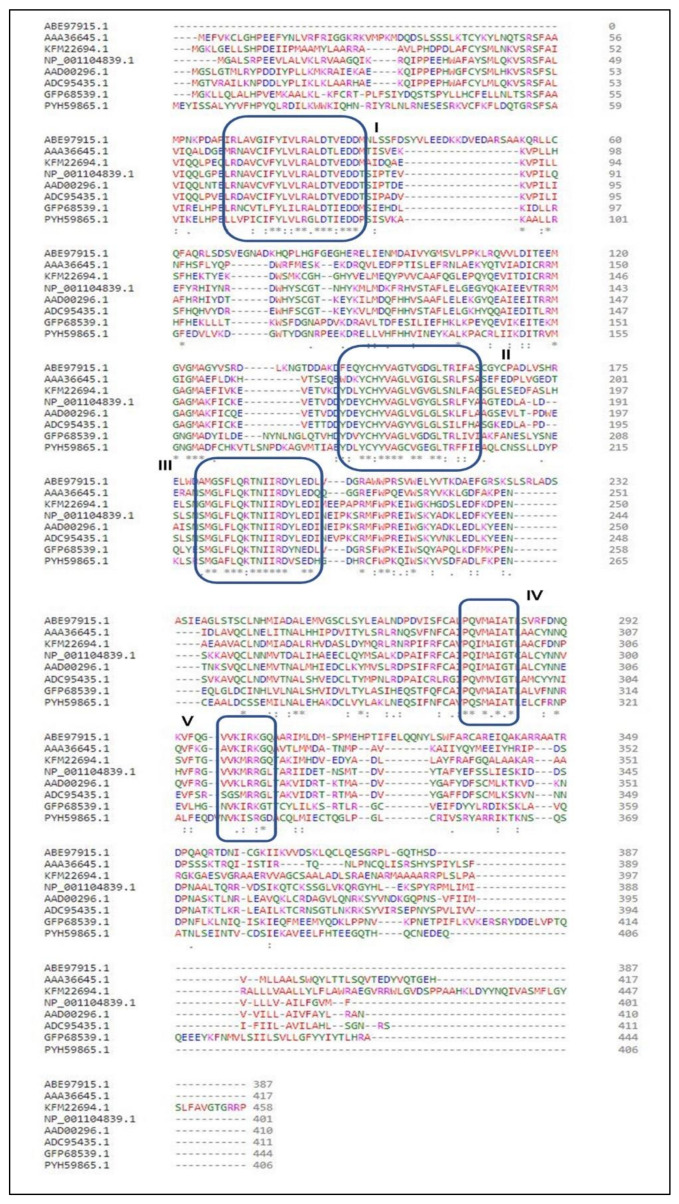
Multiple sequence alignment analysis for SQS belonging to different species: the eight sequences used in the analysis are of squalene synthase; *Aurantiochytrium limacinum* SR21 (ABE97915.1), *Homo sapiens* (AAA36645.1), *Auxenochlorella protothecoides* (KFM22694.1), *Zea mays* (NP_001104839.1), *Arabidopsis thaliana* (AAD00296.1), *Withania somnifera* (ADC95435.1), *Saccharomyces cerevisiae* (GFP68539.1), *Aspergillus niger* (PYH59865.1). The identical and similar amino acids are mentioned using dots and asterisk signs.

Conserved domain (CD) search performed for the selected protein sequence confirmed the identity of the protein as squalene synthase belonging to Isoprenoid_Biosyn_C1 superfamily (cl00210; https://www.ncbi.nlm.nih.gov/Structure/cdd/cddsrv.cgi?ascbin=8&maxaln=10&seltype=2&uid=cl00210/, accessed on 29 November 2021), as shown in Figure 1. CD-search revealed information about the enzyme, such as substrate binding pocket, Substrate-Mg^2+^ binding site, aspartate rich regions (1 and 2) indicating the deduced amino acid sequence can code for a functional enzyme. The enzymes in the isoprenoid biosynthesis class 1 superfamily contain similar ‘isoprenoid synthase fold’. The SQS enzyme generally requires NAD(P)H and Mg^2+^ for its functioning. SQS gene belonging to *Aurantiochytrium* sp. KRS101, with a 99.2% similarity of the selected gene for the study, was cloned and expressed in *E. coli* BL21. Recombinant SQS obtained was able to convert FPP to squalene in the presence of NADPH and Mg^2+^, and the product was further confirmed by GC-MS analysis [15]. The Multiple sequence alignment (MSA) analysis was performed by clustal omega using SQS sequences belonging to a different group of organisms (obtained from NCBI), such as humans, yeast, algae, plant, and fungi. Figure 2 shows the presence of five different highly conserved domains (I–V). Similarities were found in the conserved domains detected with the previous report for SQS from different microorganisms [15,16,17]. Conserved domain I was detected in the sequence for the amino acids (AA) 9 to 29 (IRLAVGIFYIVLRALDTVEDD). The aspartate-rich region 1 as found in domain I, DTVED (AA 24 to 28) was found to have similarities with human squalene synthase (AA 80 to 84) and was predicted for substrate binding [15,18,19]. Conserved domain II was detected in the sequence for the amino acids 145 to 162 (YCHYVAGTVGDGLTRIFA). Motif search tool with hidden Markov model revealed the information about the presence of Pfam id as SQS_PSY motif (position AA 08 to 303 of protein sequence, I-E-Value: 9.2 × 10^−25^). Signature motif YCHYVAGTVGDGLTRI for squalene synthase on conserved region II was found at a position of AA 145 to 160 in the sequence. Conserved domain II was also identified as an active site for SQS reported in the previous literature [16]. AA sequence from position 180 to 197 was identified as conserved domain III, which was previously recognized as a catalytic region [16]. From AA position 192 to 200 (RDYLEDLVD) in the selected SQS AA, the sequence was found to be aspartate-rich region 2. The role of these regions (Aspartate rich 1 and 2) is well characterized as a binding site for phosphate groups of the prenyl acceptor via a magnesium salt bridge [20]. The second aspartate-rich region containing DYLED (AA 193 to 200), which matches the human squalene synthase (AA 219 to 223) is the aspartate-rich region. Any mutation in the region AA 219 to 223 led to the loss of function of the enzyme. Thus, it is mainly responsible for the inactivation of the enzyme activity [17,18]. Conserved domain IV region PQVMAIATL (AA 277 to 281) was also found in the selected sequence, which was earlier predicted to be the sequence for NAD(P)H binding and catalyzing the second reaction of the squalene synthesis [17,21]. VKIRK (AA 299 to 303) conserved region V was also found similar to the human squalene synthase that is important for binding to NAD(P)H [18]. A region rich in the hydrophobic amino acids, which may contribute to transmembrane activity, was missing in the selected SQS sequence, in contrast to what was observed in many other species, which was also supported by TMHMM analysis (Section 2.3) [22]. The absence of the transmembrane (TM) region in the selected SQS may suggest improved solubility and activity [23]. This was verified by deleting the TM region (containing the hydrophobic amino acids) of *Magnolia officinalis* squalene synthase by genetic engineering resulting in improvement of the solubility and expression [23].

### 2.2. Physicochemical Characteristics In-Silico Analysis

Physicochemical characteristics for the selected gene were predicted using the ProtParam analysis tool, and the calculated values were as shown in Table 1. The SQS sequence selected for analysis consists of ORF (Open-Reading Frame) of 1161 nucleotides in length encoding 387 amino acids. The gene sequence for SQS in *Aurantiochytrium* sp. KRS101 was identified to be of 1551 bp, with two exons and an intron (1164 bp encoding 387 amino acids) [15]. The estimated molecular weight of SQS was 43.28 kDa that was falling within the range of SQS from different species, which is 40–50 kDa with certain exceptions [17]. Isoelectric point (pI) is the pH value at which the overall net charge on the protein is zero (Zwitter-ion form). The theoretical value of pI was predicted to be 5.17. The value of pI has a very large range for SQS originating from different sources and is predicted to be as high as 8.20 in some cases [24] and as low as 6.64 [17]. The values of negatively charged and positively charged amino acid residues were 56 and 43, respectively. This information is helpful during enzyme purification as well as to understand the surface topology of the protein [25]. The total number of atoms predicted in the protein was 6029. The protein extinction co-efficient at 280 nm in water was predicted to be 1.028, and it provides information regarding the estimation methods using spectrophotometry. The value of the half-life for the selected protein was 30 h, which means it will take 30 h to vanish half the proteins after its synthesis. The half-life predicted was similar to those SQS sequences found in the plant species previously [26]. The protein instability index was 41.82, showing that the protein was not stable (value higher than 40). The aliphatic index was 86.72, which consists of a basic indication of aliphatic side chain amino acids (leucine, valine, alanine, and isoleucine). The GRAVY score was −0.256, indicating that the protein is nearly non-polar in nature (value close to 0) [27]. There were 28 phosphorylation sites (15 Serine, 10 Threonine, and 4 Tyrosine residues) predicted using netphos 3.1 (https://services.healthtech.dtu.dk/service.php?NetPhos-3.1, accessed on 8 December 2021, Supplementary Materials Figure S1). The phosphorylation sites predicted are very important as they are related to the active state of the enzyme upon phosphorylation using different kinases [28]. The analysis for prediction of glycosylation sites using NetNGlyc 1.0 showed two sites for possible glycosylation (Asp 31 and As134) based on Asn-Xaa-Ser/Thr sequon presence (Supplementary Materials Figure S2). However, the analysis further revealed that although it contained the sequon, it may not be exposed to the N-glycosylation site as there is no signal peptide sequence detected in the selected sequence (Section 2.4). In general, all this information will help for designing future experiments for cloning or other modification of the SQS found in the *A. limacinum* species for overproduction of squalene.

### 2.3. Transmembrane Domain and Hydropathy Analysis

**Figure 3:**
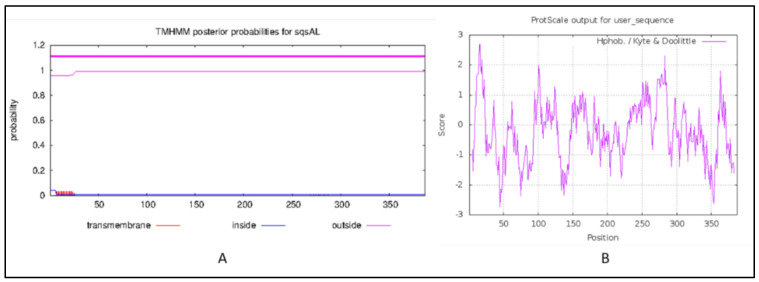
(**A**) Transmembrane helices prediction, (**B**) hydropathy analysis: The red lines on the *X*-axis indicates the probability of TM regions. *Y*-axis indicates the probability of occurrence.

The Transmembrane (TM) helices prediction performed using TMHMM 2.0 (https://services.healthtech.dtu.dk/service.php?TMHMM-2.0, accessed on 6 December 2021) revealed that the squalene synthase from *A. limacinum* did not contain any TM helices (Figure 3A), while two TM helices were reported in most of the squalene synthase that was analyzed for other species, such as plants. The absence of TM region, particularly in the SQS of *Aurantiochytrium* species, indicates the location and solubility of the enzyme in the thraustochytrids may differ from most of the SQS reported from other groups [29]. In-vitro studies about the effects of an absence of TM region on the expression and enzyme activities need to be further investigated. The hydropathy analysis performed using protscale was in the range between −2.733 and +2.678 and window size 9, as shown in Figure 3B.

### 2.4. Signal Peptide and Peroxisome Targeting Signal Prediction

**Figure 4:**
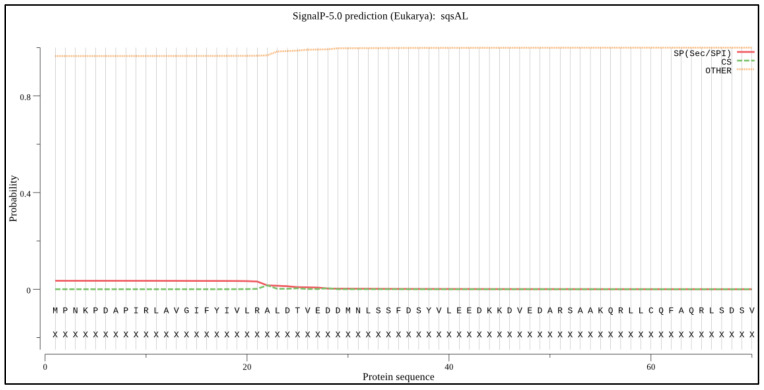
Signal peptide prediction: The *X*-axis shows the position of the amino acid sequence, the *Y*-axis indicates the probability of occurrence.

The signal peptide prediction was performed using SignalP 5.0 (https://services.healthtech.dtu.dk/service.php?SignalP-5.0, accessed on 28 November 2021). The output from the analysis, as shown in Figure 4, shows different scores for amino acid sequences. As shown in Figure 4, no signal peptide was predicted with an above-threshold value in the selected sequences, which were similar to previous studies [27]. The peroxisome targeting signal 1 (PTS1; SKL AA position 369 to 371) and 2 (PTS2; AA position 327, 330, 344, and 348) were observed at the C-terminal domain as previously identified for SQS from *Aurantiochytrium* sp. KRS101 as a possible anchor to the membrane [15].

### 2.5. Secondary Structure Prediction and Analysis

**Figure 5:**
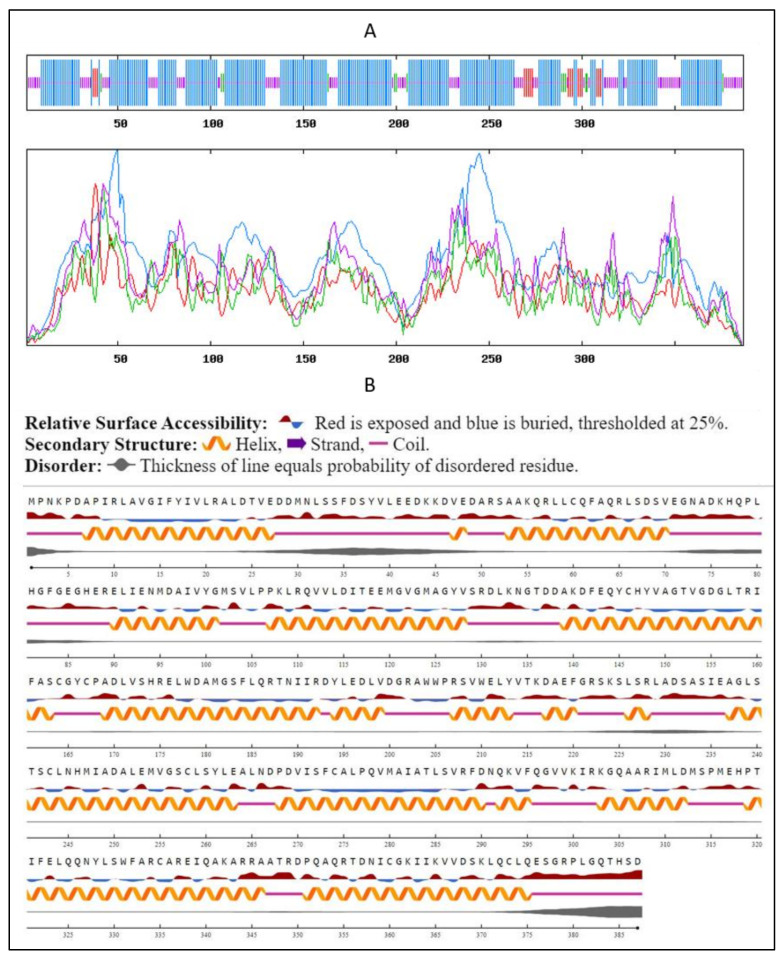
Secondary structure prediction using (**A**) SOPMA-NPSA and (**B**) NetsurfP 2.0 method: Predicted secondary structure of sqs, where blue lines indicate α-helix, the purple lines indicate random coils, the red lines indicate extended strands, and the green lines indicate β-strands.

Secondary structure prediction was performed using the SOPMA-NPSA method (https://npsa-prabi.ibcp.fr/cgi-bin/secpred_sopma.pl, accessed on 29 November 2021). It revealed that the SQS from the *A. limacinum* species is mainly composed of α-helix (66.14%) followed by random coils (25.84%), extended strands (4.39%), and β-Sheets (3.36%) (Figure 5A). Our findings were matching in the same range with a slight variation with the secondary structure predicted previously for the SQS of different species, such as *Withania somnifera* species, *Gienseng* species and species belonging to *Fabaceae* family [16,26,30,31]. These data were supported along with information about the Psi-phi dihedral angles and surface accessibility using NetsurfP 2.0 (https://services.healthtech.dtu.dk/service.php?NetSurfP-2.0/, accessed on 8 December 2021). The prediction regarding the relative surface accessibility and disorder residues was as shown in Figure 5B.

### 2.6. 3-D Structure Modeling and Analysis

**Figure 6:**
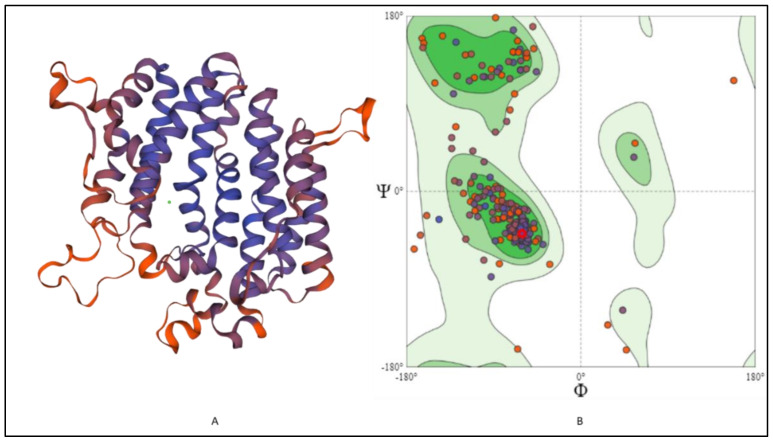
(**A**) 3-D structure modeling and (**B**) Ramachandran plot for the SQS in *A. limacinum* sp.: Showing the 3D structure of SQS predicted by SWISS-MODEL using appropriate template 3vj9.1.A (crystal structure of human SQS) and prediction of amino acids in favored regions using Ramachandran plot.

**Figure 7:**
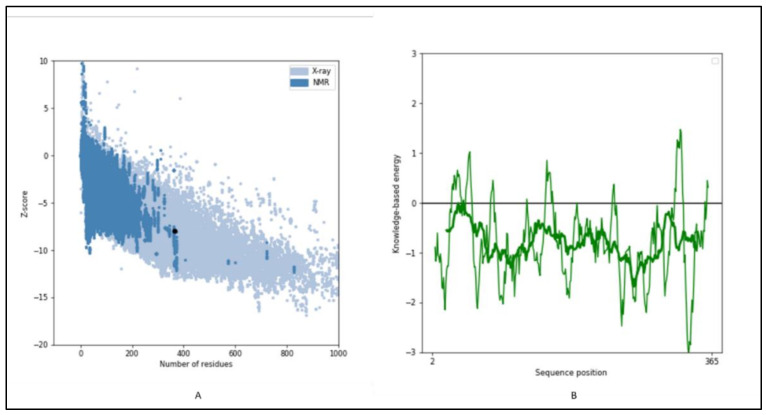
(**A**) Prosa analysis for the modeled structure of SQS, (**B**) energy plot for all AA residues: Showing the *Z*-score of energy for overall model quality.

The 3-D structure modeling was performed using appropriate templates. Nine different templates (3vj9.1.A, 3wce.1.B, 3v66.1.A, 3vja.1.A, 3wca.1.A, 3vj8.1.A, 3vja.1.A, 3vja.1.A, and 3weg.1.A) with lower e-value, higher identity, and availability of X-ray crystal structure were selected for the model prediction on SWISS-MODEL platform (http://swissmodel.expasy.org/, accessed on 16 December 2021). One model out of nine was finalized based on the Q-Mean score (−4.18) predicted based on the template structure (3vj9.1.A; crystal structure of human squalene synthase). The 3-D structure predicted was observed as shown in Figure 6A. The 3-D model generated SQS of selected species was predicted to majorly contain α- helixes and was made of a monomer with large space in the center as a binding site. The active site at the center provides space to interact with NAD(P)H and Mg^2+^ via hydrogen bonding [32]. The 3-D structure was similar to many predicted models reported earlier for the SQS from different species [16,17,26,30]. The Ramachandran plot, as shown in Figure 6B, showed that 93.37% of the model structure was in favored regions, and the protein was stereo-chemically stable. The dark green in the graph indicates the allowed regions. The stereochemical specificity and dihedral angles [ψ, ϕ] were indicated in Figure 6B. The modeled structure was further assessed by the ProSa-web server by analyzing the protein folding energy. The Z score for the SQS structure modeled was found to be −7.93. *Z*-score is the estimation based on the energy calculation for each of the amino acids and the comparison with the known template structure. The two different colors, as shown in Figure 7A, indicate the groups of structures explained by X-ray or NMR (Nuclear Magnetic Resonance). The value of the *Z*-score of modeled structure for SQS (*A. limacinum* species) was in the acceptable range (+10 to −10, a preferably negative value is good) [33]. Figure 7B shows the energy calculation for each amino acid, with all values predicted to be negative. Amino acid residues resulting in positive values were absent in the predicted SQS model [26]. H-bond energy using the VADAR analysis showed the value of −1.5 against the expected value of −2.0.

### 2.7. Prediction of Anticipated Binding Sites in SQS

**Figure 8:**
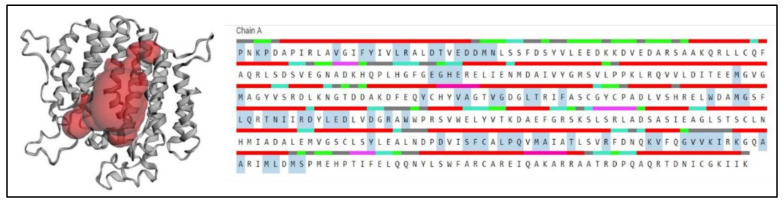
3-D structure prediction of the binding site using CASTp analysis: Showing the predicted sited of binding using CASTp servers. The amino acids highlighted in blue color indicate the predicted active site residues of SQS.

Active and binding sites were predicted using CASTp servers (http://sts.bioe.uic.edu/castp/index.html, accessed on 2 December 2021). The predicted binding sites in the SQS from *A. limacinum* are shown in Figure 8. The amino acids highlighted in blue color show the AA taking part in the formation of the binding site at the center of the protein. The binding site prediction for the SQS matched a previous report [26]. According to the analysis done with the 3D ligand site, it was observed that four sites were predicted for ligand binding for Mg^2+^ (Glu27, Asp28, Tyr145, and TRP204), which was also supported by the CD analysis, as mentioned in Section 2.1.

## 3. Materials and Methods

### 3.1. Retrieval of the Sequence Data:

The sequences for SQS gene available for all the thraustochytrids species were retrieved from NCBI (https://www.ncbi.nlm.nih.gov/, accessed on 29 November 2021) and UniProtKB database (https://www.uniprot.org/uniprot/, accessed on 29 November 2021). The respective four sequences were as mentioned in Table 2 with the basic information provided. The sequence for SQS from *Aurantiochytrium limacinum* SR21 (ATCC-MYA-1381) (previously known as *Schizochytrium limacinum*) was selected for further analysis (NCBI ID: DQ464066.2, UniProtKB id: ABE97915.1).

### 3.2. Physicochemical Characteristics Prediction

MOTIF search tool (http://www.genome.jp/tools/motif, accessed on 28 November 2021) using PROSITE database was used for motifs identification. The physicochemical characteristics, such as isoelectric point (pI), half-life, and GRAVY (Grand average of hydropathy) analysis, were obtained using the ProtParam tool of ExPasy (https://web.expasy.org/protparam/, accessed on 28 November 2021) [34]. The selected sequence was analyzed for the presence of transmembrane domains using TMHMM analysis tool (http://www.cbs.dtu.dk/services/TMHMM/, accessed on 6 December 2021) [29]. Analysis of hydrophobic and hydrophilic properties of membrane-spanning regions of the protein was performed by protscale (http://web.expasy.org/cgi-bin/protscale/protscale.pl, accessed on 28 November 2021) [26]. Analysis and identification of signal peptide sequence were performed using SignalP 5.0 (http://www.cbs.dtu.dk/services/SignalP/, accessed on 28 November 2021) [35]. Phosphorylation sites were predicted using netphos 3.1 (https://services.healthtech.dtu.dk/service.php?NetPhos-3.1, accessed on 8 December 2021) [24]. Possible N-glycosylation sites were predicted using NetNglyc-1.0 (https://services.healthtech.dtu.dk/service.php?NetNGlyc-1.0, accessed on 9 December 2021) [24]. The sequence was also checked for the possible presence of Peroxisome targeting signal (PTS) by the presence of PTS1 ((S/A/C)-(K/R/H)-(L/A) amino acids consensus sequence) and PTS2 ((R/K)-(L/V/I)-XXXXX-(H/Q)-(L/A/F) (where X is any amino acid) consensus sequence) motif at C-terminal domain [15]. Multiple sequence alignment (MSA) analysis was done using clustal omega (https://www.ebi.ac.uk/Tools/msa/clustalo/, accessed on 3 December 2021).

### 3.3. Secondary and 3-D Structure Modelling

Secondary structure prediction was performed using NPSA-SOPMA (Self-optimized Prediction Method with Alignment) (https://npsa-prabi.ibcp.fr/cgi-bin/secpred_sopma.pl, accessed on 29 November 2021) [16,36]. Psi-phi dihedral angles and surface accessibility analysis were performed using NetSurfP-2.0 (https://services.healthtech.dtu.dk/service.php?NetSurfP-2.0/, accessed on 8 December 2021) [37]. The 3-D structure modelling for the selected sequence was performed by SWISS-MODEL using appropriate template selection (http://swissmodel.expasy.org/, accessed on 16 December 2021). Protein folding energy scores for the modeled structures were predicted using prosa web servers by uploading the PDB files obtained from the SWISS-MODEL prediction (https://prosa.services.came.sbg.ac.at/prosa.php, accessed on 16 December 2021) [38]. Tertiary protein analysis was done by VADAR tools (http://vadar.wishartlab.com/, accessed on 16 December 2021).

### 3.4. Binding Site and Ligand Binding Prediction

Appropriate binding sites were predicted using CASTp servers (http://sts.bioe.uic.edu/castp/index.html?2 accessed on 2 December 2021) [39]. The ligand binding sites were also predicted using 3D ligand site (https://www.wass-michaelislab.org/3dlig/, accessed on 2 December 2021) [40].

## 4. Conclusions

In the present study, theoretical modeling and bioinformatics analysis for the squalene synthase found in the thraustochytrid species *A. limacinum* was performed. The sequence homology revealed that selected SQS could have all the important conserved domains for its regular functions and binding to substrate and co-factors. The physicochemical properties predicted will be helpful during enzyme purification as well as to understand the surface topology of the protein. Secondary and 3-D structure models predicted will be useful in future enzyme-substrate interactions using docking and computational tools. Experimental studies, such as cloning of putative gene sequence, are further required to confirm the functioning of the SQS. Detailed analysis of genes, such as SQS, could help in rational designing of possible future biotechnological interventions for a beneficial purpose, such as improved squalene production using thraustochytrids.

## Figures and Tables

**Table 1:** Physicochemical characteristics of the squalene synthase of *Aurantiochytrium limacinum*.

UniProtKB Number	Isoelectric Point (pI)	Total Number of Atoms	No. of Negatively Charged Residues	No. of Positively Charged Residues	Estimated Half-Life (h)	Aliphatic Index	Instability Index	Extinction Co-Efficient	Grand Average of Hydropathicity (GRAVY)
ABE97915.1	5.17	6029	56	43	30	86.72	41.82	44515	−0.256

**Table 2:** List of available squalene synthase genes among thraustochytrids.

Name of the Organism	UniProtKb Number	Length of AA Sequence	Molecular Mass (kDa)	Taxonomic Identifier (NCBI)
*Schizochytrium limacinum* SR21	ABE97915.1	387	43.284	87102
*Aurantiochytrium limacinum*	Q1KNJ1	387	43.284	87102
*Aurantiochytrium* sp. TA4	A0A0M4QHX0	387	43.285	1513508
*Aurantiochytrium* sp. Qe4	A0A0M5LMP0	387	43.285	1716546
*Aurantiochytrium* sp. KRS101	U3KZM8	387	43.285	797128

## Data Availability

Not Applicable.

## Supplementary Materials

The following supporting information can be downloaded at: https://www.mdpi.com/article/10.3390/md20030180/s1. Figure S1: Predicted possible phosphorylation sites in the SQS; Figure S2: Predicted possible N-glycosylation sites in the SQS.
