# Supplementary material for: Structural and Molecular Characterization of Squalene Synthase Belonging to the Marine Thraustochytrid Species Aurantiochytrium limacinum Using Bioinformatics Approach
Source: Mar Drugs. 2022 Feb 28;20(3):180. doi: 10.3390/md20030180 (PMC8955342; doi:10.3390/md20030180)
Supplement: Supplementary file 1 [file marinedrugs-20-00180-s001.zip › marinedrugs-1564391-supplementary.pdf]

Supplementary materials

Structural and molecular characterization of squalene synthase belonging to the marine thraustochytrid species *Aurantiochytrium limacinum* using bioinformatics approach.

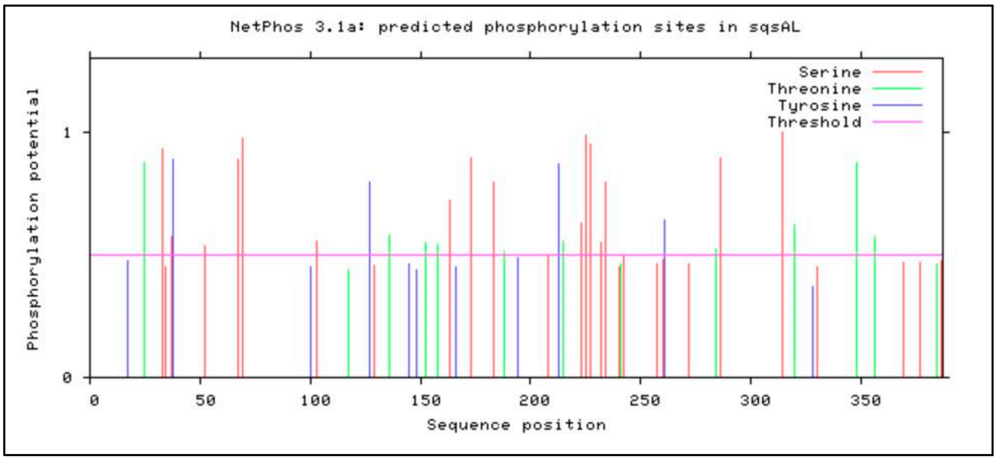

Figure S1. Predicted possible phosphorylation sites in the SQS.

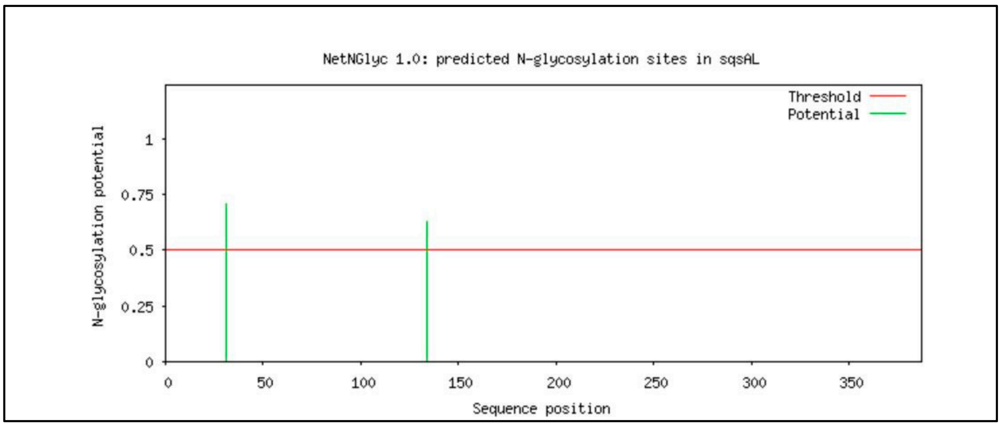

Figure S2. Predicted possible N-glycosylation sites in the SQS.
